# Qualitative Proteomic Profiling of *Saccharomyces cerevisiae* E1 Strain During Alcoholic Fermentation of Yellow Passion Fruit: A First Approximation

**DOI:** 10.3390/foods14111856

**Published:** 2025-05-23

**Authors:** Roger Consuegra-Rivera, Juan J. Román-Camacho, Inés M. Santos-Dueñas, Teresa García-Martínez, Juan Carlos Mauricio, Isidoro García-García

**Affiliations:** 1 Department of Agricultural Chemistry, Edaphology and Microbiology, Microbiology Area, Agrifood University of Córdoba, Campus of International Excellence ceiA3, 14014 Cordoba, Spain; z92corir@uco.es (R.C.-R.); b32rocaj@uco.es (J.J.R.-C.); mi2gamam@uco.es (T.G.-M.); mi1gamaj@uco.es (J.C.M.); 2 Department of Inorganic Chemistry and Chemical Engineering, Chemical Engineering Area, Institute of Chemistry for Energy and Environment (IQUEMA), University of Cordoba, Agrifood Campus of International Excellence ceiA3, 14014 Cordoba, Spain; ines.santos@uco.es; 3 Faculty of Basic and Biomedical Sciences, Center for Research and Innovation in Biodiversity and Climate Change (Adaptia), University Simón Bolívar, Barranquilla 08002, Colombia

**Keywords:** *Saccharomyces cerevisiae*, passion fruit wine, proteomics, LC-MS/MS

## Abstract

Yellow passion fruit provides a substrate suitable for alcoholic fermentation and holds valuable sensory and nutritional properties that support its use for producing wine. Among the different interesting aspects to consider in the winemaking process, we highlight the analysis of the proteins of the yeast or yeasts responsible for the process; in addition to providing fundamental knowledge about the biotransformations that take place, they can contribute to understanding basic aspects that affect the sensory properties of the wine obtained. This study aimed to analyze the proteomic profile of the yeast *Saccharomyces cerevisiae* strain E1 (ATCC: MYC-425) during passion fruit fermentation. The process was conducted in a 5 L Sartorius bioreactor with a diluted fruit puree and sucrose, making a medium with a 10% *v*/*v* alcohol potential; after 4 days, ethanol reached 8.5% *v*/*v* as sugars decreased. Subsequent LC/MS-MS analysis allowed identifying a total of 938 valid proteins: 454 from the fruit substrate and 484 attributed to yeast. Of the latter, 243 proteins were present throughout the fermentation, with GO term analysis highlighting “binding” (78%) and “catalytic activity” (66%) in molecular function, as well as “cellular” (98%) and “metabolic (90%) processes” in biological process domains. These findings may be a significant step forward in understanding the proteomic dynamics of *S. cerevisiae* in tropical fruit fermentations. By revealing key proteins and their roles in the unique conditions of passion fruit must, this study offers insights for optimizing the wine elaboration procedures and improving organoleptic properties and quality.

## 1. Introduction

Due to its geographical conditions, Colombia is not a prominent grape-producing country, which has traditionally resulted in national wines of lower quality compared to international standards. This limitation highlights the need to explore alternative raw materials, such as tropical fruits, to create value-added products that can stimulate and enhance the agro-industrial sector and compete in the national and global markets. In this context, the alcoholic fermentation of tropical fruits has undergone significant diversification, driven by a growing demand for products with unique and exotic sensory profiles [1,2].

Colombia is the third-largest producer and the leading exporter of yellow passion fruit (*Passiflora edulis* f. *flavicarpa* O. Deg) worldwide, a fruit appreciated for its exotic flavor, nutritional properties, and antioxidant activity [3]. While much of the production is dedicated to fresh consumption or low-value-added products, including pulps, juices and concentrates, the introduction of a passion fruit alcoholic beverage, such as wine, may offer a significant opportunity to diversify its applications, enhance the production chain, and contribute to rural agro-industrial development. Additionally, the use of surplus passion fruit to produce new fermented products offers a long-term solution to overproduction, waste reduction, and resource efficiency, contributing to a circular economy. Such innovation could generate economic opportunities in the agricultural sector and expand export markets with a high-quality functional product [4,5,6,7,8].

Passion fruit wine is an alcoholic beverage that, although under development, is renowned for its unique aroma and flavor profile derived from the fruit’s exotic characteristics. However, its high acidity and predominantly artisanal production methods present challenges [9]. Although scientific research on passion fruit wine is limited, studies have explored fermentation methods, aromatic properties, and innovative formulations to enhance its implementation and appeal [10,11,12,13,14]. The development of this product requires a deep understanding of the biochemical and molecular mechanisms involved in fermentation, where the activity of the yeast plays a pivotal role in determining the wine’s sensory quality [15,16].

Proteomics, the study of proteins in complex biological systems, is fundamental to understanding the biochemical processes in alcoholic fermentation and their impact on the final product’s quality [10,11]. The proteomic characterization of *Saccharomyces cerevisiae* in fruit fermentations is particularly relevant, as protein profiles can vary significantly depending on the substrate and fermentation conditions, directly influencing the development of aromas and flavors characteristic of wine [12,13,14,15]. Several investigations highlight the diverse applications of proteomic analysis in understanding *S. cerevisae* behavior in various fermentation contexts. One research effort examines the proteomic changes in a native *S. cerevisiae* strain cultivated in grape must, revealing that proteins involved in amino acid metabolism, glycolysis, and sterol formation dominate during the early fermentation stages, showcasing the yeast’s metabolic adaptations [16]. Another investigation examines the protein profiles of a flor yeast *S. cerevisiae* strain and a conventional yeast strain, identifying the proteins responsible for producing volatile compounds during the sparkling wine fermentation process. This analysis emphasized the role of proteomics in linking protein expression to the sensory attributes of sparkling wines [17]. A complementary study provided a comprehensive evaluation of the biological processes in *S. cerevisiae* P29, a sparkling wine strain, during the second fermentation under two distinct conditions: with and without endogenous CO_2_ overpressure. Proteomic data collected at the middle and final stages offered valuable insights into the yeast response to different environmental pressures, contributing to a deeper understanding of its role in the production of high-quality sparkling wines [18]. Together, these investigations underscore the utility of proteomic tools in uncovering the molecular mechanisms of *S. cerevisiae*, enhancing our ability to optimize fermentation processes across several products.

In recent years, proteomic research applied to oenology has advanced considerably. Several studies have shown that *S. cerevisiae* plays an essential role in ethanol production and the synthesis of secondary metabolites, such as esters and aldehydes, which contribute to the sensory properties of wine [17,19,20]. Several factors influence the proteome abundance of *S. cerevisiae*, with the chemical composition of the substrate playing an important role in the quantity of adaptive proteins [21,22,23]. Equally important, fermentation conditions—such as pH, temperature, and nutrient availability—have a significant impact on the yeast’s proteomic profile and, ultimately, the quality of the finished product [24,25,26,27]. Identifying proteins that persist throughout the fermentation process can provide valuable insights into the stability of volatile compounds and their impact on the wine’s sensory properties [18,28].

Omics tools have transformed the study of yeast in the production of wine and other fermented products, offering comprehensive insights into their metabolic and functional dynamics. Among these, proteomics stands out, particularly techniques based on liquid chromatography coupled with tandem mass spectrometry (LC-MS/MS) [29,30]. LC-MS/MS is a powerful analytical tool that enables the sensitive and precise identification and quantification of proteins in biological samples. Its broad dynamic range and ability to analyze complex proteomes without the need for prior isolation make it especially advantageous for in situ studies, providing detailed information about the composition and behavior of the microbiota throughout fermentation processes [31,32]. In the context of winemaking and related fermentations, LC-MS/MS has proven invaluable for uncovering metabolic pathways and adaptive responses of yeast under specific conditions. For example, it has been used to analyze differential protein expression in *S. cerevisiae* strains under environmental stress, shedding light on their performance during alcoholic fermentation [33].

Recent advances in yeast proteomics applied to wine and fermented beverages have reinforced the role of LC-MS/MS in characterizing metabolic pathways and aroma compound biosynthesis. Notable contributions include the profiling of aroma-active proteins in passion fruit wine [34], metaproteomic studies in vinegar fermentations [35], and functional mapping of yeast proteins in white wine production [10]. Furthermore, recent work by Ye et al. has demonstrated how different fermentation methods influence the quality attributes and microbial diversity of passion fruit wine, emphasizing the importance of microbial dynamics and fermentation strategies in tropical fruit substrates [9]. Building upon this, Zhao et al. have provided an in-depth review of yeast protein functionality, structural features, and analytical approaches, consolidating the relevance of proteomics in understanding fermentation behavior and its impact on product quality [11]. Collectively, these findings support the relevance and novelty of the present study within the growing field of tropical enology. This study aims to analyze the proteomic profile of *S. cerevisiae* (strain E1, ATCC: MYC-425) during the alcoholic fermentation of passion fruit, using LC-MS/MS to identify key proteins involved in the process. The findings may provide fundamental insights into the molecular mechanisms underlying passion fruit fermentation and establish a scientific basis for optimizing the sensory quality of passion fruit wine. This research contributes to filling a knowledge gap in the proteomics of tropical fruit fermentations and provides a foundation for developing optimization practices in passion fruit wine production, strengthening Colombia’s agro-industrial competitiveness in the global market.

## 2. Materials and Methods

### 2.1. Physicochemical Characterization of the Fruit

The yellow passion fruit (*Passiflora edulis* f. *flavicarpa* O. Deg) used in this study was grown in tropical regions of Colombia and imported by a national supplier to the facilities of the University of Córdoba (Córdoba, Spain). The fruit pulp is derived from a matrix of producers located in various tropical regions of Colombia, such as Huila, Tolima, Valle del Cauca, and Santander, which are known for their role as principal cultivators and exporters of yellow passion fruit in the country. The ripe fruit pulp was provided in 1 kg plastic packages in a frozen homogeneous puree, seedless, preservative-free, and with 0.1% ascorbic acid added as an antioxidant. The pulp was kept at −20 °C in the lab and thawed first in the refrigerator and then at room temperature to maintain the fruit’s nutritional value and sensory qualities. The fruit was physicochemically characterized in triplicate using an electronic hydrostatic balance (COBOS JT-300C, Madrid, Spain) to measure density, a portable digital refractometer (HANNA HI 96800, Woonsocket, RI, USA) to measure total soluble solids, a pH meter (CRISON Basic 20, Barcelona, Spain) to determine pH, and an acid-base titration with 0.25 M NaOH to determine titratable acidity [36].

### 2.2. Must Preparation

**Figure 1:**
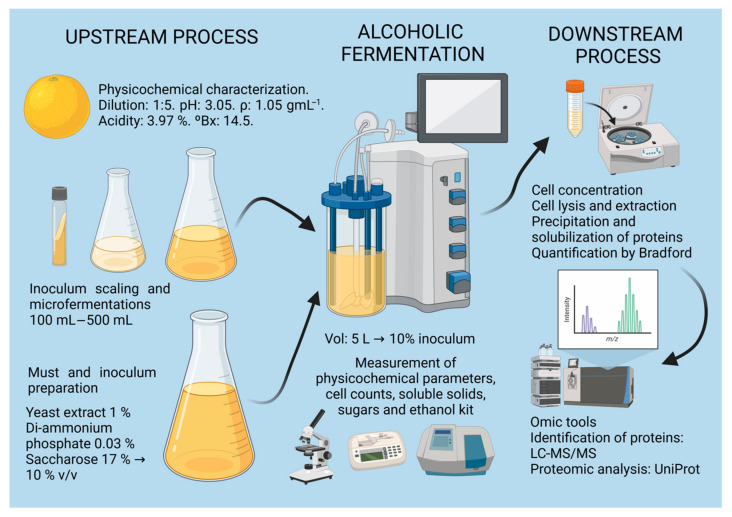
Passion fruit wine proteomics workflow. The upstream process includes the physicochemical characterization of the fruit, the scaling of the inoculum, and the preparation of the must; alcoholic fermentation refers to the production of wine within the bioreactor, and the downstream process refers to the proteomics of the final product. Created with BioRender.com (https://www.biorender.com).

The high acidity of passion fruit has substantial implications for winemaking. This element may represent a challenge in terms of sensory balance, as excess acidity can produce wine with an extremely acidic or even astringent organoleptic profile, affecting consumer acceptance. Pulp dilution and sugar addition are common ways to mitigate these effects [34,37]. The fruit pulp was diluted in a 1:5 ratio with distilled water, and sucrose was added to reach a final concentration of 170 g/L, yielding a theoretical alcohol potential of 10% *v*/*v*. Additionally, 1% yeast extract and 0.03% diammonium phosphate (DAP) were incorporated as sources of readily assimilable nitrogen to optimize the growth and metabolic activity of *S. cerevisiae*. 4.5 L of the must was prepared for each passion fruit wine production batch, maintaining the same proportions as in the 500 mL working culture and ensuring consistency between the inoculum and the reactor-scale fermentation. The preparation of the must and the sequential steps of the methodology are summarized in Figure 1.

### 2.3. Yeast Strain and Inoculum Preparation

The yeast strain *Saccharomyces cerevisiae* E1 (ATCC: MYC-425) was selected for the alcoholic fermentation of yellow passion fruit. Originally isolated from the Montilla-Moriles region in Andalucía, Spain, this strain was stored at −80 °C in a 20% (*v*/*v*) glycerol solution as a cryoprotectant in the Microbiology Laboratory of the Department of Agricultural Chemistry, Edaphology, and Microbiology at the University of Córdoba. *S. cerevisiae* was selected because it is widely recognized as the most used yeast species for fruit wine fermentation [9,38,39,40,41,42].

Inoculum preparation followed a stepwise approach to scale up the initial culture volume, ensuring efficient adaptation and a rapid start to fermentation [43]. Each step included a 48 h incubation at 28 °C. The pre-inoculum was prepared by thawing *S. cerevisiae* E1 and streaking it on inclined YPD agar (10 g/L yeast extract, 20 g/L peptone, 20 g/L dextrose, and 20 g/L agar). The starting inoculum was then prepared by culturing the pre-inoculum in 100 mL of YPD broth. Next, 100 mL of this culture was transferred to 400 mL of must, prepared following the same composition previously described, including diluted fruit pulp, sucrose, yeast extract, and DAP. This mixture resulted in a 500 mL working culture, representing 10% of the total fermentation volume in the bioreactor.

### 2.4. Alcoholic Fermentation

The fermentation protocol followed methodologies previously established by the research group [44,45,46]. The biotransformation of the fruit was conducted in batch mode using stirred-tank bioreactors of 5 L (Sartorius Biostat-Aplus, Göttingen, Germany) equipped with probes for control and monitoring pH, agitation, dissolved oxygen, and temperature, suitable for laboratory-scale fermentations. Each fermentation run used an average working volume of 5 L, with a 10% inoculum (500 mL), an agitation speed of 300 rpm, and a temperature of 28 °C.

The biotransformation process was monitored over 4 days. Wine samples were collected at key time points (2, 24, 48, 72, and 96 h) from bioreactors. Several variables were recorded at each sampling point, and physicochemical measurements were taken. These included pH, titratable acidity, determined via acid-base titration with 0.25 M NaOH, and total soluble solids, measured with a portable digital refractometer [36]. Sugars and ethanol concentrations were quantified using Megazyme^®^ enzymatic kits: K-GLUC for glucose, K-FRUGL for fructose, K-SUFRG for sucrose, and K-ETOH for ethanol [47]. Total cell counts (cells/mL) were determined with a Neubauer chamber. Also, plate cultures for each sample were carried out to check that the only active microorganism in the process was the one inoculated.

### 2.5. Proteomics

#### 2.5.1. Sampling and Cell Concentration

The proteomic protocol was based on metaproteomic investigations in vinegar production that the research group had previously published; this procedure has been applied for the first time to the passion fruit wine matrix [35,48,49].

**Figure 2:**
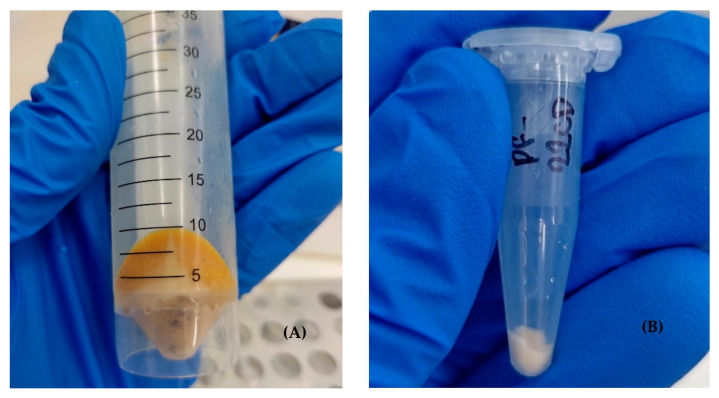
(**A**) Sample collection and cell concentration process. Centrifugation was performed to eliminate plant fragments from the substrate that adhered to yeast cells, ensuring a cleaner sample for further analysis. (**B**) Pellet formation after microtube centrifugation, illustrating the separation of yeast cells from residual plant material.

Samples were collected from the bioreactor every 24 h in triplicate during the four days of fermentation. From each sample, 200 mL was taken and distributed in four Falcon centrifuge tubes of 50 mL. Cells of the wine samples were collected by centrifugation at 2400× *g* for 10 min at 4 °C (Hettich, Rotina 38R, Westphalia, Germany) followed by washing with 40 mL of cold sterile distilled water and centrifugation again at 2400× *g* for 5 min at 4 °C to remove the vegetal remains of the fruit adhered to the cells, see Figure 2A.

Pellets were resuspended in 600 µL of cold sterile distilled water and the homogenate was transferred to microtubes and centrifuged twice at 16,900× *g* for 30 s at 4 °C (Eppendorf, 5418 R, Hamburg, Germany). Finally, the pellet was resuspended in 600 µL of Phosphate Buffered Saline (PBS), the supernatant was discarded, and the cell concentrate was stored at −80 °C until further processing, see Figure 2B.

#### 2.5.2. Cell Lysis and Protein Extraction

The cell pellets were resuspended in 600 μL of an extraction buffer containing 100 mM Tris-HCl (pH 8.0), 2 mM dithiothreitol (DTT), 1 mM ethylenediaminetetraacetic acid (EDTA), and 1 mM phenylmethylsulfonyl fluoride (PMSF), supplemented with a Protease Inhibitor Cocktail (Roche, Basel, Switzerland) as per the manufacturer’s instructions. Cells were subjected to mechanical lysis by shaking with glass beads (Sigma-Aldrich, St. Louis, MO, USA) in a Vibrogen Zellmühle at cycles of 1 min of shaking and 1 min on ice (10 cycles in total). Glass beads and cell debris were removed by sequential centrifugation: first at 16,900× *g* for 20 min at 4 °C, and second at 4200× *g* for 10 min at 4 °C. The supernatant was recovered and stored at −20 °C for the next phase.

#### 2.5.3. Protein Precipitation and Solubilization

Proteins were precipitated by incubating the supernatant overnight at −20 °C with four volumes of ice-cold 10% (*w*/*v*) trichloroacetic acid (TCA)-acetone-DTT. The precipitated proteins were collected by centrifugation at 14,000× *g* for 50 min at 4 °C. The resulting pellets were washed twice with 500 μL of ice-cold 0.07% (*w*/*v*) acetone-DTT, stored at −20 °C for 30 min, and centrifuged again at 16,900× *g* for 15 min at 4 °C. The protein pellets were vacuum dried using a SpeedVac™ concentrator (Eppendorf 5301, Hamburg, Germany).

The dried protein pellets were solubilized in 500 μL of buffer of 8 M urea, 2% (3-[(3-cholamidopropyl) dimethylammonio]-1-propanesulfonate) (CHAPS), and 20 mM DTT at 1200 rpm and 4 °C for four cycles of 30–45 min using a Heidolph™ Vibramax 100 shaker (Schwabach, Germany). Solubilized samples were centrifuged at 16,900× *g* for 15 min at 4 °C.

#### 2.5.4. Protein Quantification

Protein concentration was determined by the Bradford method, using a BSA (bovine serum albumin) standard with dilutions of 0.25 to 1.00 µg/µL; absorbances were measured at 595 nm [50].

#### 2.5.5. LC-MS/MS Analysis

Proteomic analysis was performed at the Research Support Central Service (SCAI), University of Córdoba, Spain, by using liquid chromatography coupled to tandem mass spectrometry (LC-MS/MS). From each sample, ≥50 μg of protein was subjected to analysis.

Proteins were separated using Sodium Dodecyl Sulfate-Polyacrylamide Gel Electrophoresis (SDS-PAGE) with a 10% polyacrylamide gel at 100 V. Protein bands were washed with 200 mM ammonium bicarbonate (AB) containing 50% and 100% acetonitrile (ACN) for 15 and 5 min, respectively. Reduction was carried out using 20 mM DTT in 25 mM AB at 55 °C for 20 min, followed by alkylation with 40 mM iodoacetamide in 25 mM AB for 20 min in the dark. After two washes in 25 mM AB, the proteins were digested overnight at 37 °C with trypsin (12.5 ng/μL, Promega, WI, USA). Digestion was stopped by adding 1% trifluoroacetic acid (TFA), and the samples were dried using a SpeedVac™ concentrator.

Peptides were analyzed using a Dionex Ultimate 3000 nano UHPLC system equipped with a C18 analytical column (Thermo Fisher Scientific, Waltham, MA, USA). The peptides were trapped on a C18 pre-column using 2% ACN/0.05% TFA at a flow rate of 5 μL/min for 5 min. Separation occurred over a 150 min gradient (4% to 90% acetonitrile with 0.1% formic acid) at 300 nL/min and 40 °C. Peptides were ionized via nanoelectrospray and analyzed using an Orbitrap Fusion mass spectrometer (Thermo Fisher Scientific, MA, USA). Survey scans were performed at 120 K resolution (400–1500 *m*/*z*), and tandem MS (MS/MS) was conducted on precursors with charge states 2–5. Dynamic exclusion was set to 15 s with a 10 ppm tolerance, and the instrument operated in top speed mode with 3 s cycles for optimal MS/MS event coverage.

Prior to the LC-MS/MS analysis, preliminary optimization trials were performed using passion fruit matrix samples. These trials focused on adjusting the acetonitrile gradient, column temperature, and MS scan parameters to enhance peptide separation, reduce background noise, and improve reproducibility. Settings were refined based on previous studies conducted by the research group on similar food matrices, such as wine and vinegar [29,35,48,51,52].

#### 2.5.6. Raw Data Analysis

The raw data from the mass spectrometry were processed using Proteome Discoverer (version 2.1.0.81, Thermo Fisher Scientific, MA, USA). MS/MS spectra were analyzed using the SEQUEST search engine against the UniProt database for protein identification of the species *S. cerevisiae* and the family Passifloraceae (http://www.uniprot.org).

Tryptic digestion was set to allow up to one missed cleavage. Carbamidomethylation of cysteines was specified as a fixed modification, while oxidation of methionines was included as a variable modification. The precursor mass tolerance was set at 10 ppm, and product ions were searched with a tolerance of 0.1 Da. Peptide Spectral Matches (PSMs) were validated using the Percolator algorithm, ensuring a False Discovery Rate (FDR) of 1%. Identified peptides were grouped into proteins based on the principle of parsimony and further filtered to maintain an FDR of 1%.

After identification, proteins with a score > 2 and with a number of peptides ≥ 2 were selected as valid proteins. Proteins that were common in two out of the three biological replicates of both inoculum and fermentation samples were selected for this purpose; an enrichment analysis was performed in the Uniprot database [53] using the Gene Ontology (GO) annotation tool (http://geneontology.org/) to find the most relevant functions and processes associated with the domains of the biological process (BP) and molecular function (MF) of the *S. cerevisiae* genome strain ATCC 204,508/S288c (Baker’s yeast).

Frequency bar charts were built using dynamic tables in Microsoft^®^ Excel (version 365, USA). Venn diagrams highlighting overlapping proteins across replicates were generated using Venny 2.1.0 (https://bioinfogp.cnb.csic.es/tools/venny/, accessed on 21 May 2025).

## 3. Results and Discussion

### 3.1. Physicochemical Properties of Yellow Passion Fruit Pulp

The physicochemical properties shown in Table 1 provide valuable information on the suitability of ripe yellow passion fruit pulp for alcoholic fermentation.

The pulp, with a thick and consistent texture, had the physicochemical variables shown in Table 1, confirming the sharp acidity typical of passion fruit as well as, through °Bx, a high sugar concentration, essential for fermentation. These values correspond to the undiluted pulp as received, prior to any dilution or must preparation steps.

These findings align with previous studies by Santos et al. [37] on the fermentation of Caatinga passion fruit. This study demonstrated that ripe fruit has a higher pH, more soluble solids, and lower titratable acidity than unripe fruit. Ramaiya et al. [54] found a titratable acidity of 1.8 ± 0.1% *w*/*v* for the purple passion fruit variety and 1.9 ± 0.1% *w*/*v* for the yellow passion fruit; while Santos et al. [55] found values of 8.4 ± 0.1% *w*/*v* for the Caatinga passion fruit and 6.3 ± 0.1% *w*/*v* for the yellow passion fruit. The titrable acidity in wine grapes ranges between 0.6% and 1.5%, depending on variety, climate, and maturity stage [56], indicating that passion fruit has a high acidity level.

In terms of sugar content, He et al. [4] reported 13.4 °Bx for purple passion fruit and 14.3 °Bx for the yellow variety, while Santos et al. [55] found 12.3 °Bx for yellow passion fruit and 13.2 °Bx for Caatinga passion fruit. The 14.5 °Bx observed in this study aligns with these previous reports, confirming that the sugar content of yellow passion fruit is within the expected range for this fruit. However, when compared to wine grapes, which typically range between 22 and 25 °Bx for red varieties [57], the sugar content of passion fruit is relatively low. This suggests that, although passion fruit can serve as a fermentable substrate, adjustments such as sugar supplementation may be necessary to achieve ethanol levels comparable to those of traditional grape-based wines.

### 3.2. Analysis of Variables Throughout the Fermentation Process

The progression of alcoholic fermentation is reflected in the interplay between sugar consumption, soluble solids, ethanol production and yeast population. Table 2 summarizes these variables, highlighting the activity of *Saccharomyces cerevisiae* throughout the process.

Throughout the fermentation process, the gradual depletion of sucrose, glucose, and fructose, combined with the decline in soluble solids (°Bx) and the rise in ethanol concentration, underscores the metabolic efficiency of *S. cerevisiae* regarding sugar consumption under these specific conditions.

Process monitoring determined that fermentation lasts 4 days, with a final alcohol content of 8.5% ± 0.4% *v*/*v*, as determined by the residual sugar in the wine. Sucrose, the primary sugar in the must and the source for glucose and fructose, showed the most pronounced reduction, particularly during the first 48 h of fermentation, indicating its rapid utilization. Ethanol concentrations increased progressively, peaking at 8.5 ± 0.4% *v*/*v* by the end of fermentation (96 h), confirming the effective conversion of sugars into alcohol. By this point, the residual concentrations of sucrose, glucose, and fructose were 17.7 g/L, 2.9 g/L, and 3.9 g/L, respectively, summing up to 24.5 g/L. This residual sugar content corresponds to the theoretical remaining potential for ethanol production (1.5% *v*/*v*), explaining the difference between the obtained ethanol concentration (8.5% *v*/*v*) and the expected maximum yield (10% *v*/*v*). By 96 h, °Brix values and sugar levels both stabilized, showing yeast’s shift into a stationary phase with low fermentative activity. The 4-day window was deemed best for recording the most active phase of fermentation, as no more major biochemical changes were noted past this point, and, thus, proteomic analysis was limited to this period.

The cell counts observed during fermentation provide important information about the yeast population’s overall progression. During the initial 24 h, total cell counts increased significantly from 3.9 ± 1.1 × 10^7^ cells/mL to 10.5 ± 1.3 × 10^7^ cells/mL, reflecting the active growth phase of *S. cerevisiae* as it utilized the abundant nutrients and sugars available in the must. After this period, total cell counts stabilized, remaining consistent between 10.4 ± 0.8 × 10^7^ cells/mL at 48 h and 11.2 ± 0.6 × 10^7^ cells/mL at 96 h. This plateau indicates that the yeast population had reached its maximum density, characteristic of a system where nutrient availability and environmental conditions limit further population growth.

These variations highlight fermentation efficiency and the yeast’s adaptation to the conditions of the passion fruit must for ethanol production [58].

Compared to previous studies, the fermentation time and alcohol yield observed in this study are consistent with reported values for passion fruit fermentation. Ye et al. [9] fermented wine from unripe and ripe purple passion fruit pulp for 20 days; the highest alcohol content recorded was 9.2 and 10.3% on the 10th day, respectively. Santos et al. [37] evaluated the quality of fermented alcoholic beverages from passion fruit produced using passion fruit species obtained from the Brazilian Caatinga biome (*Passiflora cincinnata* Mast.). The fermentative process took six days, and the alcohol content ranged from 7.0 to 8.1% *v*/*v*.

### 3.3. Proteomic Analysis

According to the previously detailed methodology, a total of 938 valid proteins were identified, of which 484 (52%) belonged to the yeast strain and the remaining 454 (48%) to the Passifloraceae family. The latter belonged to the yellow passion fruit plant, the fermentation substrate, which has not been analyzed in more detail to avoid confusion in the functional interpretation of the yeast. Proteomic analysis was performed for the 484 proteins of *S. cerevisiae*. These proteins were identified through peptide fragmentation and sequence alignment using the SEQUEST algorithm with the UniProt database. No predefined structural biomarkers were used for classification.

**Figure 3:**
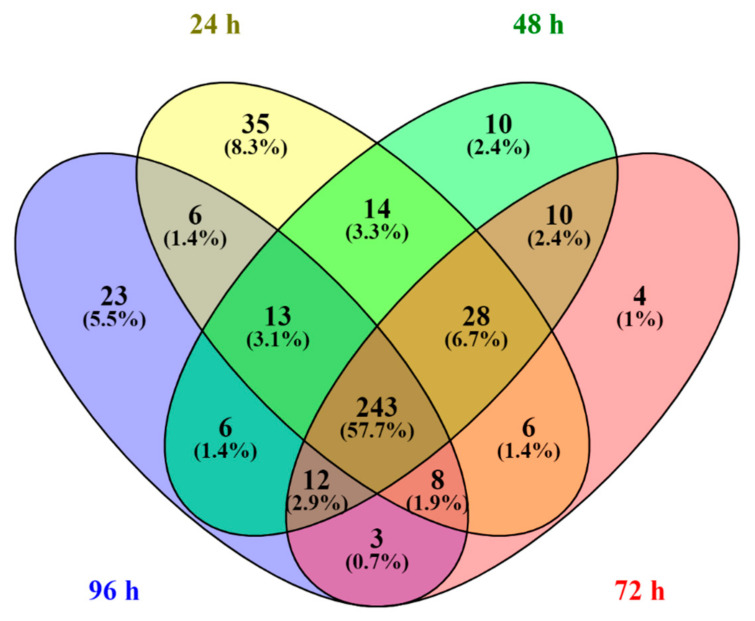
Venn diagram showing the number of common and specific proteins found at each sampling point of alcoholic fermentation (24, 48, 72, and 96 h). Values in brackets indicate the proportion of the total number of common proteins (421) of *Saccharomyces cerevisiae* E1 (ATCC: MYC-425).

To ensure data reliability and research focus, proteins found in at least two of the three biological replicates for each sample point (24, 48, 72, and 96 h) were included. This refinement reduced the dataset to 421 out of 484, consistently valid proteins, which provided the most accurate proteomic data during fermentation. Of these, 353 proteins were found at 24 h, 336 at 48 h, and 314 at 72 and 96 h. Among the 421 common proteins, 35 (8.3%) were specific to 24 h, 10 (2.4%) to 48 h, 4 (1%) to 72 h, and 23 (5.5%) to 96 h. The analysis highlighted a core group of 243 proteins (57.7%), present at all sampling points, suggesting a robust and stable metabolic network throughout the alcoholic fermentation (see Figure 3 and File S1, Supplementary Materials).

The findings showed a modest decline in proteins throughout fermentation. However, this does not necessarily suggest a decrease in the concentration or activity of yeast cells. Instead, it likely reflects adjustments in the proteomic profile of *S. cerevisiae* in response to the evolving conditions of the fermentation environment. These adaptations might be associated with the yeast’s metabolic response to ethanol-induced stress, nutrient depletion, or a shift toward pathways prioritizing survival and maintenance over active growth [16,59]. The stabilization of total cell counts after 48 h suggests that, after an initial phase of active growth, *S. cerevisiae* may have entered a stationary phase, where cellular adaptations could have played a role in sustaining metabolic activity under increasing ethanol stress and nutrient limitations.

### 3.4. GO Term Enrichment Analysis

**Figure 4:**
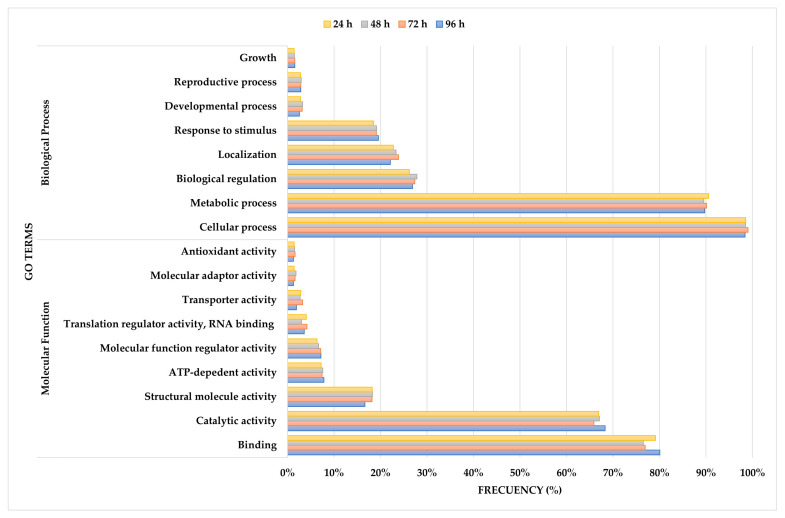
GO term enrichment analysis of the proteins found at each sampling time (24, 48, 72, and 96 h), represented as the frequency (%) of proteins associated with each GO Term at the second hierarchical level within the “biological process” and “molecular function” domains.

**Figure 5:**
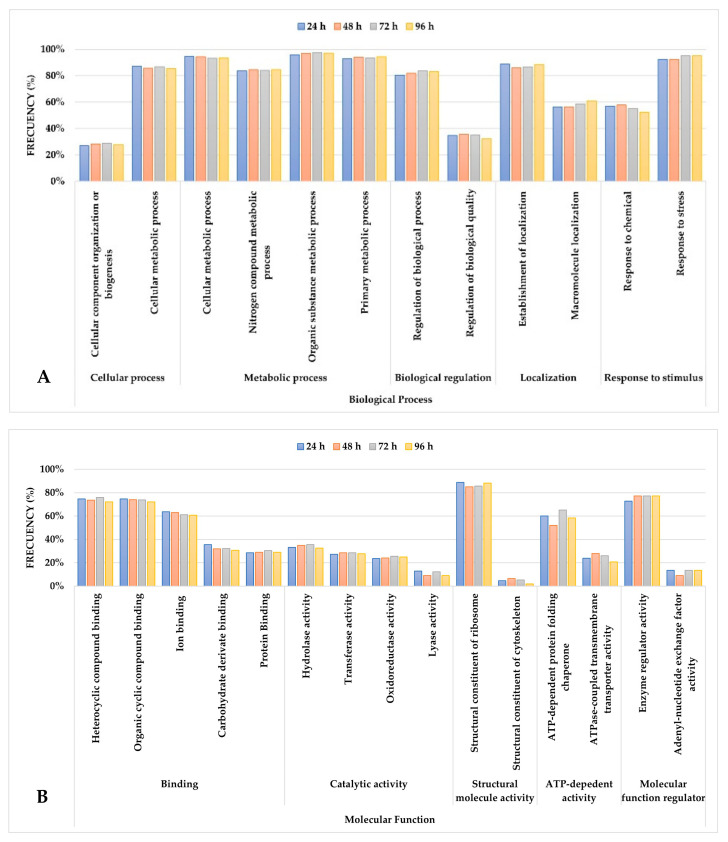
(**A**) Summary of the most represented GO terms at the third hierarchical level within the “biological process” domain. (**B**) Representation of the same dataset at the third hierarchical level within the “molecular function” domain.

GO term enrichment analysis was performed for common proteins (421) found at 24, 48, 72, and 96 h to identify biological processes and molecular functions involved in the adaptation of *S. cerevisiae* to passion fruit fermentation; this analysis provides insights into the yeast’s metabolic response throughout the alcoholic transformation process. GO terms with a frequency ≥1% were categorized within the “biological process” and “molecular function” domains, emphasizing key roles and adaptation of the yeast to the conditions of this tropical substrate. At the second hierarchical level, the most prevalent GO terms provided a broad classification of the metabolic functions active during fermentation, emphasizing essential cellular processes and enzymatic activities (Figure 4). Further analysis at the third hierarchical level refined these findings, revealing more specific GO terms associated with key proteins throughout fermentation. These were grouped into the “biological process” domain (Figure 5A) and the “molecular function” domain (Figure 5B), offering a more detailed view of the molecular mechanisms at play. Although fluctuations in GO term frequencies were observed at different sampling points, the variations were minimal, suggesting that the core metabolic functions remained relatively stable throughout fermentation. These slight changes may reflect adjustments in metabolic priorities as *S. cerevisiae* responded to the progressive depletion of nutrients and increased ethanol concentration [16,59].

Some of the proteins identified in this study, as shown in Table 3, are key markers in the metabolic processes that promote alcoholic fermentation, including glycolysis, ethanol production, and the formation of aroma and flavor compounds. These results complement the analysis shown in Figure 4 and Figure 5, providing additional context regarding specific proteins involved in biological processes and molecular functions highlighted in the “biological processes” and “molecular functions” domains. Proteins, such as alcohol dehydrogenase (adh1) and pyruvate decarboxylase (pdc1), play a central role in converting sugars to ethanol while generating intermediates that fuel other essential processes [60]. Additionally, enzymes involved in secondary metabolite synthesis contribute to the formation of volatile compounds that are important in determining the aroma profile of the wine [28,61]. A complete list of GO terms and associated proteins identified in this study can be found in Supplementary Materials File S1. These results may provide a clearer view of the molecular processes involved in fermentation and their role in shaping the properties of the final product.

#### 3.4.1. Biological Process Domain

The “biological process” domain contains the following broad groups of GO terms: “cellular process”, “metabolic process”, “biological regulation”, “localization”, and “response to stimulus”. Within this domain, it can be observed how cellular metabolism is present during all fermentation, meaning the permanence of great enzymatic activity to convert the sugars into ethanol. A frequency > 98% in the “cellular process” was consistent during fermentation and reflected the growth capacity that *S. cerevisiae* must maintain and adapt to the conditions of passion fruit must [9,62].

“Metabolic process”, with a frequency of approximately 90%, points to the continuous activity of the central metabolic routes, including glycolysis, alcoholic fermentation, and biosynthesis of secondary metabolites. These activities ensure not only the energy supply of the cell but also precursors for volatile compounds to be synthesized that are responsible for the aromatic profile of the wine as a final product [60,63].

Dephosphorylation of phosphoenolpyruvate leads to the formation of pyruvate, which is then decarboxylated to acetaldehyde by the sequential activity of the central proteins pyruvate kinase (pk1) and pdc1, which further leads to the production of ethanol. At the same time, the formation of NADH molecules required for reducing acetaldehyde to ethanol occurs when glyceraldehyde-3-phosphate dehydrogenase (tdh3) acts [60,64], see Table 3. This activity is essential for producing levels of alcohol in developing wine, constituting an essential step in volatile compound formation and thereby enriching the aromatic profile [38,42]. From the above, the processes of glycolysis are a way of ensuring the energy supply obtained from the fermentable sugar intake of the passion fruit must.

The GO term “biological regulation” (~27%) states that it is indicated by the activation of a feedback mechanism that is used for the optimization of metabolic pathways. Additionally, post-translational modification by protein phosphorylation can regulate metabolic enzymes in such a way that their activity is either increased or decreased, thereby providing rapid adjustment to signals of internal or environmental origin, respectively. The proteomic analysis of *S. cerevisiae* in this study revealed the presence of enzymes regulated by phosphorylation/dephosphorylation, including acetyl-CoA carboxylase (acc1), pyruvate kinase (cdc19), fructose-1,6-bisphosphatase (fbp1), glutamate dehydrogenase (gdh1), hexokinase (hxk1), and malate dehydrogenase (mdh1). These proteins, identified among the 421 common proteins, play key roles in the following metabolic regulation: acc1 is involved in fatty acid biosynthesis, cdc19 regulates glycolysis by catalyzing the conversion of phosphoenolpyruvate to pyruvate, fbp1 participates in gluconeogenesis, gdh1 is crucial for amino acid metabolism, hxk1 catalyzes the phosphorylation of hexoses in the first step of glycolysis, and mdh1 is essential for the tricarboxylic acid (TCA) cycle and redox balance. These enzymes contribute to the yeast’s adaptation to fermentation conditions and have also been referenced in previous studies, highlighting their relevance in yeast metabolism [65,66,67]. Further details on these proteins, including their specific GO terms and functional roles, are provided in the Supplementary Materials (File S1).

The GO Term “localization” (~23%) refers to the transport, spatial distribution, and organization of molecules, organelles, and proteins within the cell. These processes are essential for maintaining cellular function during alcoholic fermentation. These mechanisms play a key role in ensuring the efficient operation of metabolic pathways, allowing *S. cerevisiae* to adapt to the conditions of passion fruit must and sustain ethanol production and aroma compound biosynthesis [68]. Among the proteins identified in this study within the “Localization”, ATPase BiP/Kar2p (kar2) was found to be particularly relevant. This protein is involved in the proper folding and transport of other proteins within the endoplasmic reticulum, a function that may contribute to cellular homeostasis under the stress conditions of fermentation. Efficient protein localization, including the role of kar2, likely supports yeast adaptation by facilitating enzymatic activity and metabolic regulation throughout the process. The ability of *S. cerevisiae* to dynamically reorganize its cellular components underpins its capacity to maintain metabolic efficiency, produce secondary metabolites, and ensure viability under changing environmental conditions [63].

The GO term “response to stimulus” (~19%) encompasses the mechanisms through which *S. cerevisiae* maintains metabolic homeostasis by detecting and responding to environmental changes, including osmotic, oxidative, and alcoholic stress. This adaptation is crucial for yeast survival and fermentation performance. Among the proteins identified in this study, trehalose-6-phosphate synthase (tps1) plays a key role in protecting cellular structures and maintaining protein stability under high ethanol concentrations [69,70]. Additionally, several proteins involved in secondary metabolite biosynthesis contribute to the formation of volatile compounds that influence the final aromatic profile of the wine. Specifically, pdc1, adh1, branched-chain amino acid transaminase (bat1), isopropylmalate synthase (leu2), and acetolactate synthase (ilv2) participate in metabolic pathways linked to the production of aroma-active compounds [40,61,71].

#### 3.4.2. Molecular Function Domain

The most abundant GO terms in the “molecular function” domain included “binding”, “catalytic activity”, “structural molecule activity”, “ATP-dependent activity”, and “molecular function regulator activity”.

High enrichment found for “binding” (76–80%) highlighted the importance of protein–protein and protein–ligand interactions in maintaining metabolic flux and cellular stability during fermentation. These interactions are critical for enzymatic activity, signal transduction, and metabolic regulation. Among the proteins identified in this study, tdh3, adh1, and enolase (eno2), were present and associated with cofactor binding and substrate recognition, supporting metabolic processes under fermentation conditions. The detection of binding-related proteins in this study aligns with previous reports on the role of cofactor interactions and structural stabilization in yeast metabolism [70,72].

The GO term “catalytic activity” (66–68%) includes enzymes that mediate essential biochemical reactions, facilitating sugar metabolism, redox balance, and energy production. Several proteins detected in this study, such as adh1, cdc19, hexokinases (hxkA, hxkG, hxk1, hxk2), fructose-bisphosphate aldolase (fba1), and glucose-6-phosphate isomerase (g6pi), are known to play key roles in glycolysis, the pentose phosphate pathway, and fermentative metabolism. The identification of these enzymes is consistent with previous studies, reinforcing their relevance in fermentation efficiency and the biosynthesis of aromatic compounds, such as esters and higher alcohols, which shape the sensory profile of passion fruit wine [61,73].

As for “structural molecule activity” (~18%), this GO term refers to proteins involved in cytoskeletal organization, ribosomal stability, and overall cellular integrity, which are essential for yeast adaptation to fermentation stress. In this study, proteins, such as ribosomal proteins (rps3, rps5) and actin-related proteins (act1, crn1), were identified, indicating their role in supporting translation efficiency and maintaining cell morphology under fermentation conditions. The presence of these structural proteins is in line with findings from similar research, emphasizing the importance of a stable translational machinery and cytoskeletal framework in yeast viability during alcoholic fermentation [59,70].

The GO term “ATP-dependent activity” (~7%) includes proteins involved in ATP-driven cellular functions, such as energy metabolism, active transport, and stress response mechanisms. The proteomic analysis identified heat shock proteins (ssa1, ssa2), ATP synthase subunits (atp1, atp2), all of which participate in protein refolding, energy production, and intracellular homeostasis. The detection of these ATP-dependent proteins aligns with previous studies, supporting their role in energy supply during fermentation and stress adaptation in yeast [60,64].

Finally, GO Term “molecular function regulator activity” (~7%) includes proteins that modulate the activity of enzymes and transporters, ensuring a coordinated response to metabolic and environmental changes. One of the regulatory proteins identified in this study was peroxiredoxin (tsa1), which plays a key role in redox homeostasis by mitigating oxidative stress caused by reactive oxygen species (ROS). The presence of tsa1 and other regulatory proteins is consistent with previous research highlighting the significance of redox balance and enzymatic regulation in yeast stress response mechanisms [21].

## 4. Conclusions

A proteomic approach using LC-MS/MS was applied to study the dynamics of alcoholic fermentation in passion fruit pulp, expanding the potential for optimizing the process to produce high-quality tropical wines. The proteomic analysis of *Saccharomyces cerevisiae* strain E1 (ATCC: MYC-425) revealed a consistent profile of proteins associated with the main physicochemical variables analyzed during alcoholic fermentation; this reflected the yeast’s ability to adapt to the unique conditions of passion fruit must, characterized by its high acidity and rich content of bioactive compounds. Although sensory attributes were not evaluated in this study, the proteomic analysis provided a valuable foundation for understanding yeast–substrate interactions and their potential impact on product quality. Additionally, the functional GO term enrichment analysis highlighted critical biological processes and molecular functions by the study of key proteins involved in cellular metabolism, catalytic activity, and binding capability, thus reinforcing the role of *S. cerevisiae* in tropical enology and its broader biotechnological applications.

In the future, the added value of such findings would then concern metabolomic analyses and studies associating quantitative proteomics with key parameters, such as ethanol tolerance and fermentative kinetics. This global approach will allow the development of more effective innovation strategies in tropical fruit winemaking to achieve higher-quality products and be competitive in global markets.

## Figures and Tables

**Table 1:** Physicochemical characterization of the ripe fruit pulp (undiluted raw material). Data shows mean values and the standard deviation (SD) of each variable.

	Variable	Value
Mean ± SD	Density (g/mL)	1.05 ± 0.01
	pH	3.1 ± 0.1
	Soluble solids (°Bx)	14.5 ± 0.1
	Titratable acidity (% *w*/*v* citric acid)	3.9 ± 0.1

**Table 2:** Main variables throughout the alcoholic fermentation process. Data show mean values and their standard deviation (SD) of variables at each sampling time (2, 24, 48, 72 y 96 h) are included.

	Variable	2 h	24 h	48 h	72 h	96 h
Mean ± SD	Sucrose (g/L)	130.1 ± 0.4	99.2 ± 9.1	54.9 ± 4.8	32.7 ± 2.4	17.7 ± 3.4
	Glucose (g/L)	7.3 ± 0.5	5.4 ± 0.7	2.9 ± 0.7	3.0 ± 0.3	2.9 ± 0.5
	Fructose (g/L)	7.5 ± 0.7	5.1 ± 0.6	3.9 ± 0.5	3.9 ± 0.3	3.9 ± 0.4
	Soluble solids (°Bx)	14.8 ± 0.1	12.4 ± 0.7	9.5 ± 0.5	7.9 ± 0.5	6.8 ± 0.4
	Ethanol (% *v*/*v*)	0.1 ± 0.1	2.1 ± 0.1	3.3 ± 0.4	7.1 ± 0.4	8.5 ± 0.4
	Cell counts (10^7^ cel/mL)	3.9 ± 1.1	10.5 ± 1.3	10.4 ± 0.8	11.1 ± 0.6	11.2 ± 0.6

**Table 3:** Metabolic pathway identified key proteins and specific functions associated with the GO terms of the proteomic profile of *Saccharomyces cerevisiae* E1 (ATCC: MYC-425) in passion fruit wine.

Metabolic Pathway	Key Proteins	Function
Glycolysis	Glyceraldehyde-3-phosphate dehydrogenase (tdh3)	Catalyzes the conversion of glyceraldehyde 3-phosphate to 1,3-bisphosphoglycerate, generating NADH
	Pyruvate kinase (pk1)	Converts phosphoenolpyruvate to pyruvate, releasing ATP
Ethanol Fermentation	Pyruvate decarboxylase (pdc1)	Decarboxylates pyruvate to acetaldehyde
	Alcohol dehydrogenase (adh1)	Reduces acetaldehyde to ethanol using NADH, regenerating NAD^+^ for glycolysis, reduces aldehydes to higher alcohols
Synthesis of Aroma and Flavor Compounds	Branched-chain amino acid transaminase (bat1)	Converts branched-chain amino acids (BCAAs) to keto-acids, precursors for higher alcohols
	Acetolactate synthase (ilv2)	Initiates the biosynthesis of valine and isoleucine

## Data Availability

Data is contained within the article or Supplementary Materials).

## Supplementary Materials

The following supporting information can be downloaded at: https://www.mdpi.com/article/10.3390/foods14111856/s1. File S1: GO term enrichment analysis of *S. cerevisiae* E1 (ATCC: MYC-425) proteins present at all sampling points during the alcoholic fermentation of yellow passion fruit.
